# Antenatal ultrasound diagnosis of huge fetal hydrometrocolpos secondary to imperforate hymen and successful postnatal treatment: a case report

**DOI:** 10.1515/crpm-2023-0019

**Published:** 2023-10-30

**Authors:** Mequanint Melesse Bicha, Zelalem Ayichew Workneh

**Affiliations:** Department of Obstetrics and Gynecology, Maternal Fetal Medicine Unit, University of Gondar, Gondar, Ethiopia; Department of Obstetrics and Gynecology, Urogynecology and Pelvic Reconstructive Surgery Unit, University of Gondar, Gondar, Ethiopia

**Keywords:** hydrometrocolpos, prenatal ultrasound, imperforate hymen, hymenectomy

## Abstract

**Objectives:**

Hydrometrocolpos is a pelvic cystic mass representing the distension of the vagina and uterus due to obstructive congenital anomalies of the female causing accumulation of fluid secretions in the vagina and endometrial cavity. Prenatal diagnosis is uncommon and usually noticed during the adolescent period for failure to see menses with cyclic abdominal pain, abdominal mass, and local compressive symptoms. Late diagnosis after delivery of newborns with this condition results in poor outcomes from local compressive symptoms.

**Case presentation:**

Here, we present a case diagnosed with congenital hydrometrocolpos at 39 weeks of gestation during routine third-trimester ultrasound scanning. The newborn was delivered vaginally and huge hydrometrocolpos secondary to imperforate hymen was diagnosed postnatally, and a hymenectomy was done and the newborn was discharged and improved from the hospital.

**Conclusions:**

Although congenital hydrometrocolpos occurs rarely, it is also better to suspect prenatally in a female fetus with a cystic pelvic mass. Antenatal ultrasound diagnosis of this condition will help to make decisions early and to prevent further complications which might occur both intrauterine and after birth.

## Introduction

Congenital hydrometrocolpos is a pathologic condition in which the uterus and vagina of a fetus or neonate are distended with sterile secretions and mucus. Hydrometrocolpos is an infrequent condition [1]. Congenital imperforate hymen is probably the most common obstructive anomaly of the female reproductive tract in which a layer of epithelialized connective tissue that forms the hymen has no opening and completely obstructs the vaginal introitus. Hydrocolpos and hydrometrocolpos can occur secondary to this condition [2]. The other possible causes are transverse vaginal septum, distal vaginal agenesis, transverse vaginal septum, and obstructed hemi-vagina [3].

The incidence of congenital imperforate hymen in term infants has been reported to be 0.1 %. Prenatal diagnosis of isolated hydrometrocolpos secondary to congenital imperforate hymen is a rare condition in prenatal ultrasound examination. It is important to prenatally confirm the presence of associated anomalies, which helps in the provision of proper counseling for the parents and planning of postnatal management [4]. Here, we report a case of congenital hydrometrocolpos secondary to imperforate hymen diagnosed antenatally with ultrasound.

## Case presentation

**Figure 1:**
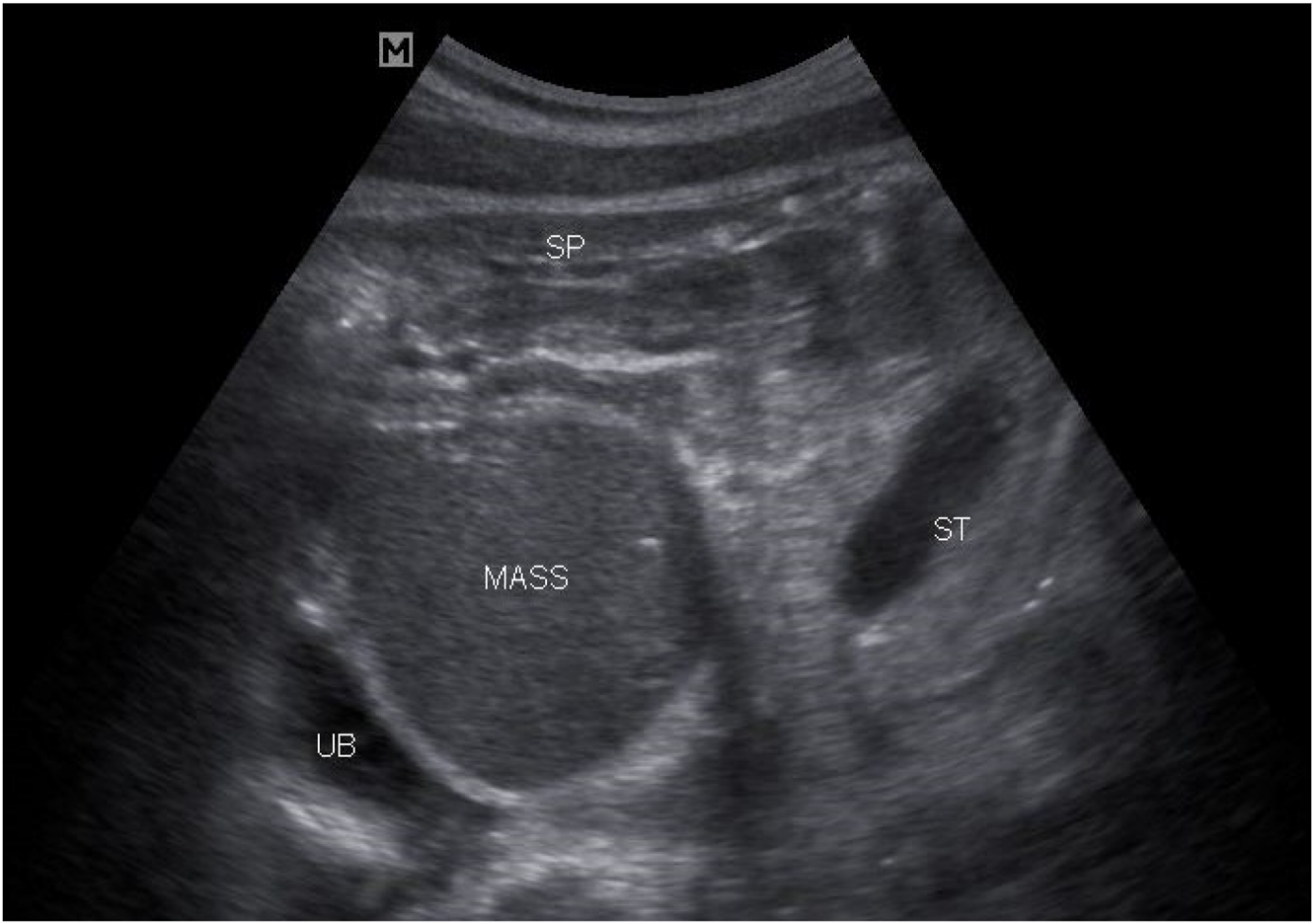
Antenatal ultrasound showing Huge retrovesical abdominopelvic cystic mass with internal echoes. UB, urinary bladder; ST, stomach; SP, spine.

**Figure 2:**
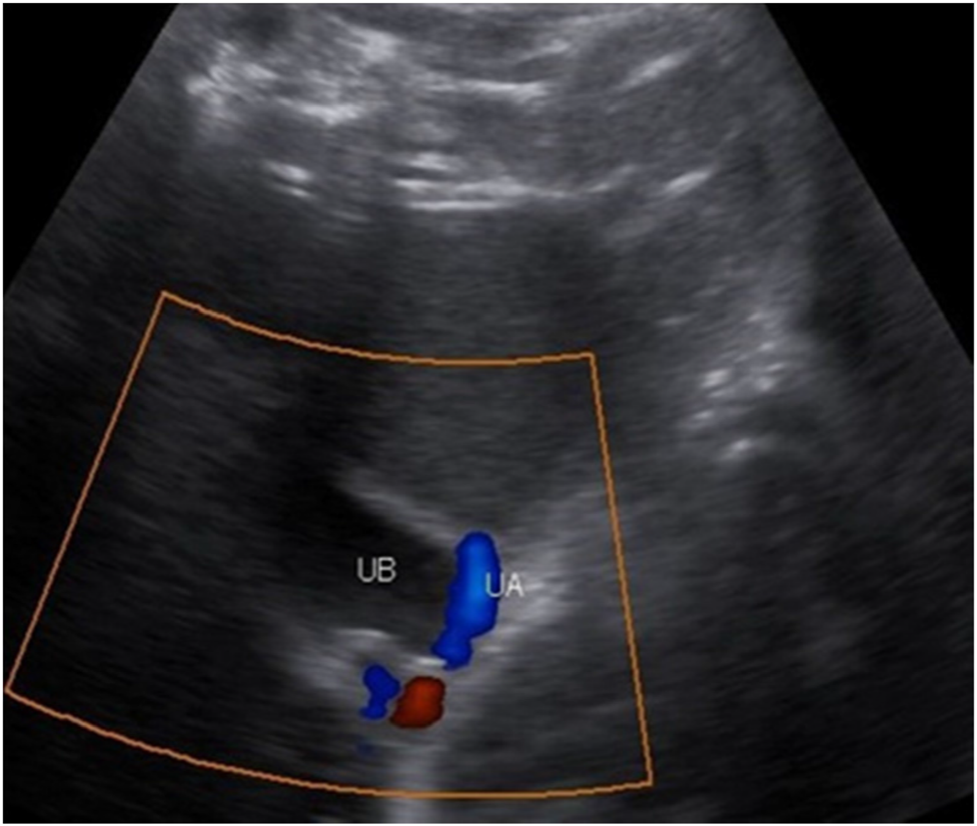
Prenatal obstetric ultrasound showing the fetal urinary bladder with peri vesical umbilical arteries. UB, urinary bladder; UA, umbilical arteries.

**Figure 3:**
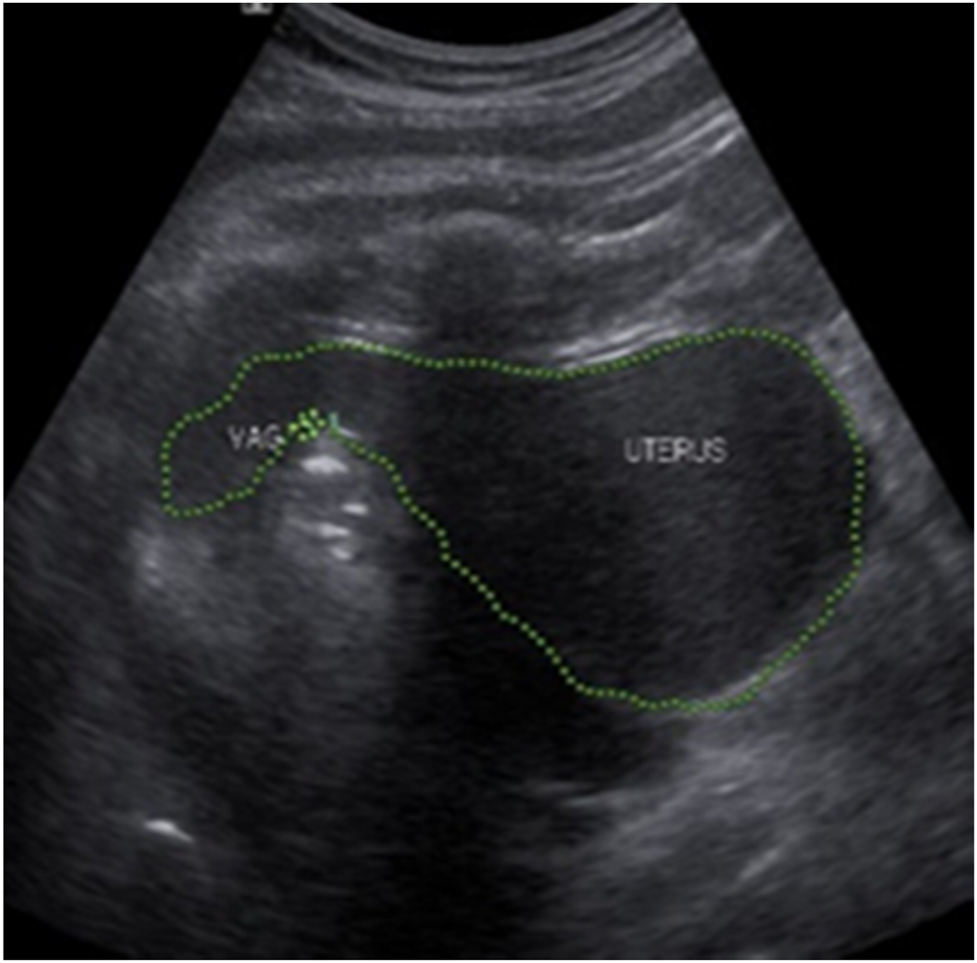
Mid-sagittal antenatal ultrasound view of the cystic pelvic mass showing hydrometrocolpos. The cystic mass is shown with line tracing.

**Figure 4:**
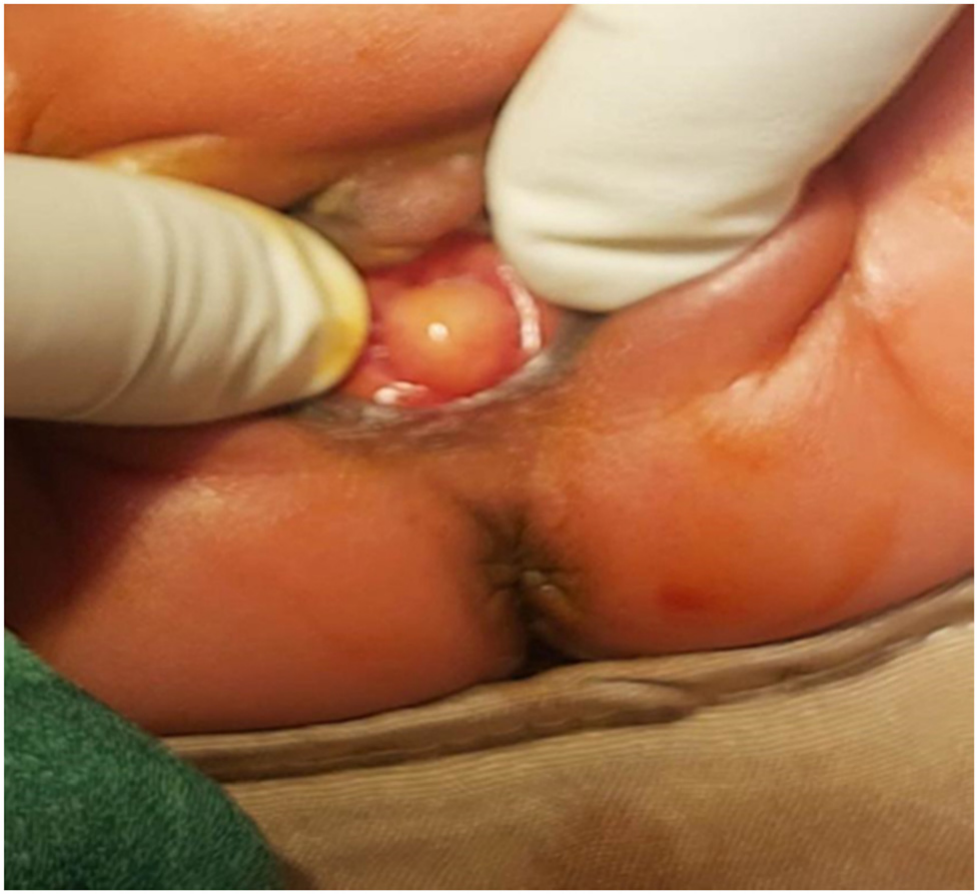
Bulged imperforate hymen.

**Figure 5:**
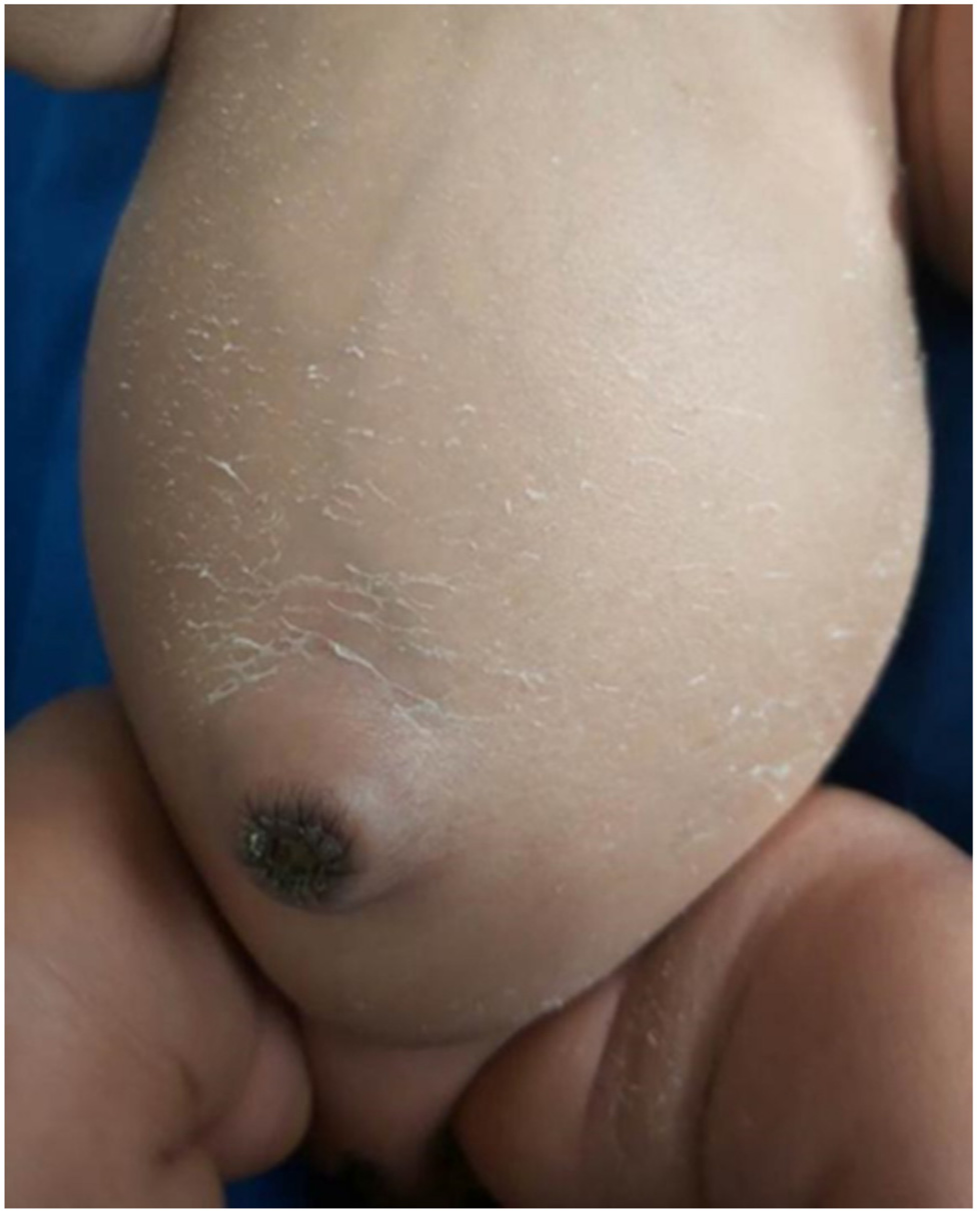
Distended abdomen of the newborn.

**Figure 6:**
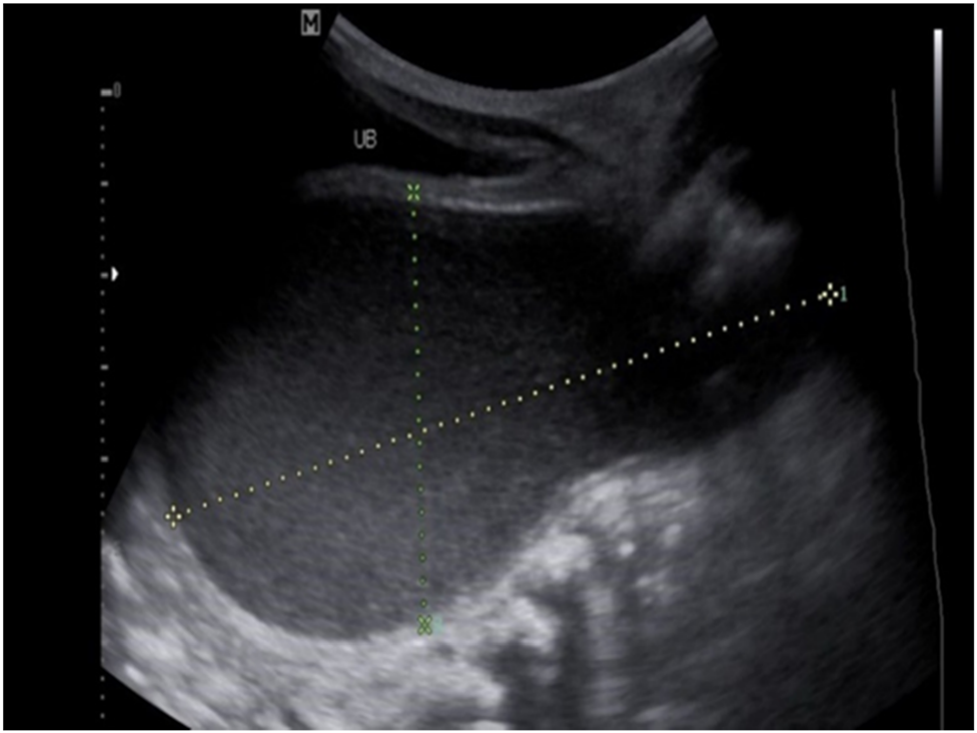
Post-delivery neonatal abdominopelvic ultrasound showing hydrometrocolpos. UB, urinary bladder.

**Figure 7:**
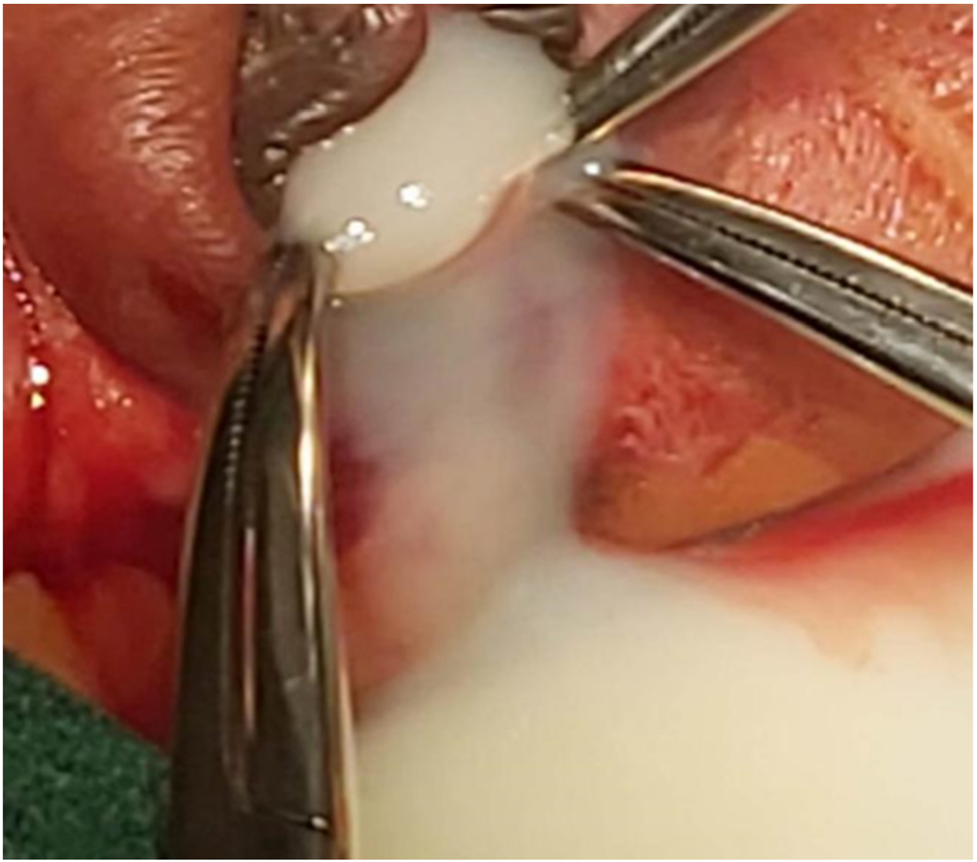
Whitish (milky) fluid was drained from the vagina and uterus.

**Figure 8:**
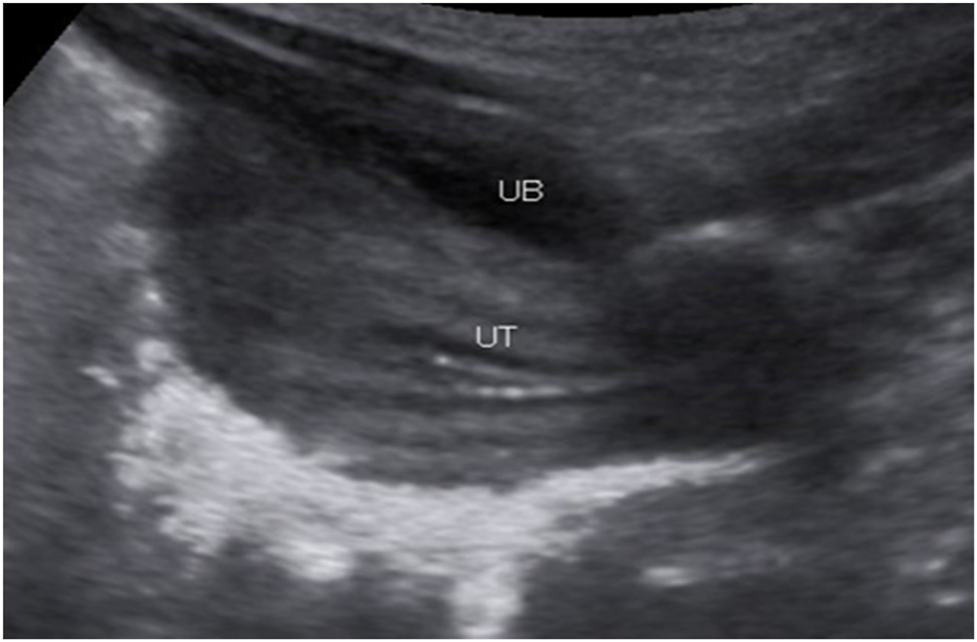
Neonatal abdominopelvic ultrasound showing empty uterus and vagina at the third post-operative day. UT, uterus; UB, urinary bladder.

**Figure 9:**
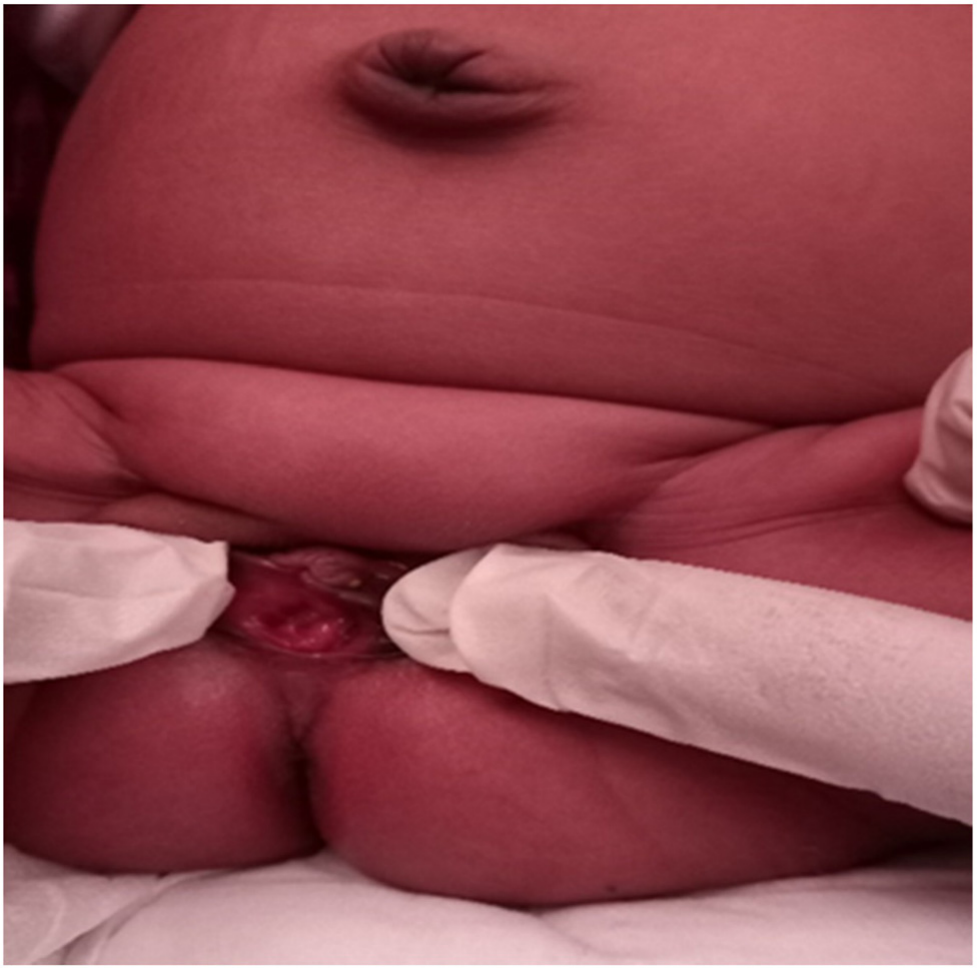
Normal vaginal orifice on the 14th postoperative day.

A 25-year old primigravid woman came to the University of Gondar Hospital fetal medicine outpatient clinic after she was referred for a fetal intraabdominal cystic mass seen on routine ultrasound scanning for a biophysical profile. Her medical and obstetric history was unremarkable. She had antenatal care and obstetric ultrasound was done at the fifth and seventh months and she was told that her fetus is in good condition. At 39 weeks of gestation, she came to us referred for advanced ultrasound scanning and we found an 80 × 60 × 40 mm retro vesical, oval, midline pelvic cystic mass with internal echoes (Figures 1, 2, and 3). There was minimal calyceal dilation of both kidneys but the urinary bladder was seen and it was having a normal outline and volume. There was no abnormality in the anatomic scanning of the other systems. The amniotic fluid volume was normal and the biophysical profile was reassuring. The woman was counseled and induction of labor was done with oxytocin and she gave birth to a 3200-g female alive newborn. Immediately upon delivery the newborn was examined and there was an imperforate hymen that bulged forwards (Figure 4) and the abdomen was distended (Figure 5), otherwise, the perineum, anus, and urethral orifice were normal. The baby passed urine and meconium. Grossly there was no dysmorphic feature in the newborn. Abdominopelvic ultrasound was done and showed a 100 × 70 × 40 mm oval cystic pelvic mass behind the bladder and there was also mild calyceal dilation of both kidneys, but there was no other abnormality (Figure 6). A renal function test was done for the newborn and found to be normal. Hymenectomy was done on the second day of delivery and around 200 mL of milky fluid was removed (Figure 7). The abdominal distention was relieved, and the renal calyceal dilation disappeared. Abdominal ultrasound after the procedure showed an empty uterus and vagina (Figure 8) and the newborn was discharged improved. The newborn came back after 2 weeks for a checkup and she was doing well with normal hymen remnants and vaginal orifice (Figure 9).

## Discussion

Hydrometrocolpos is uncommon in the perinatal period. It occurs when a genital tract obstruction is associated with the accumulation of cervical and endometrial gland secretions from the fetus’s cervical mucinous glands stimulated by maternal estrogen in the presence of an imperforate hymen. Imperforate hymen is not the only cause of hydrometrocolpos. Transverse vaginal septum is also reported to be a cause of hydrometrocolpos causing obstructive symptoms in the neonatal period [5].

The prenatal differentiation of female urogenital anomalies can be difficult because of their rarity, variations in presentation, and poor imaging by ultrasonography, especially in late gestation. Hydrometrocolpos may be mistaken for an abdominal cystic mass. The possible differential diagnoses are ovarian cyst, duplication cyst, mesenteric cyst, meconium cyst, urachal cyst, anterior meningocele, and a pelvic component of sacrococcygeal teratoma. Antenatal MRI can also be done to further delineate the lesion and to demonstrate the relationship to adjacent structures. The communication between uterine and vaginal components was better seen with Magnetic Resonance Imaging (MRI) than with sonography due to its superior contrast resolution [6]. In this case, Prenatal ultrasound was enough to diagnose hydrometrocolpos which was confirmed to be caused by congenital imperforate hymen after delivery.

As a complication, imperforate hymen during the perinatal period may lead to intraabdominal calcifications [7], fetal ascites [8], or obstructive uropathy. In our case, no complications were seen except mild calyceal dilation of the renal pelvis and abdominal distention which was improved after the hymenectomy. Most of the time, an imperforate hymen is an isolated finding, but polydactyly, duplication of the ureter, ectopic ureter, urethral membrane, imperforate anus, multicystic dysplastic kidney, and bifid clitoris have been reported [2]. There was no associated finding in our case.

Asymptomatic congenital imperforate hymen can be managed expectantly without early surgical intervention. It is also possible to have a spontaneous opening of an imperforate hymen in conservatively managed infants [9]. However, if the hydrometrocolpos is large enough to cause obstructive symptoms, hymenectomy is indicated [4, 6, 9, 10]. In our case, we did a hymenectomy because the mass was big to cause abdominal distention for prevention of progressive obstructive uropathy and renal failure.

## Conclusions

Although congenital hydrometrocolpos occurs rarely, it is also better to suspect prenatally in a female fetus with a cystic pelvic mass. Detailed prenatal scanning is recommended and management of the etiology of the obstruction should be managed after delivery of the newborn. Antenatal ultrasound diagnosis of this condition will help to make decisions early and to prevent further complications which might occur both intrauterine and after birth.
